# The efficacy of haemoperfusion combined with continuous venovenous haemodiafiltration in the treatment of severe viral encephalitis in children

**DOI:** 10.1186/s13052-023-01411-0

**Published:** 2023-02-15

**Authors:** Jun-Lin Zhao, Zhi-Yuan Wang, Shu-Jun Li, He-Kai Ma, Xue Liu, Xiao-Wen Zhan, Wei-Wei Niu, Peng Shen

**Affiliations:** Department of Pediatrics, The First Affiliated Hospital of Xinxiang Medical University, No. 88 of Jiankang Road, Weihui, 453100 Henan Province China

**Keywords:** Neopterin level, Blood purification, Haemoperfusion

## Abstract

**Background:**

This study investigated the efficacy of the integrated blood purification mode of early haemoperfusion (HP) combined with continuous venovenous haemodiafiltration (CVVHDF) in children with severe viral encephalitis, and evaluated the correlation of cerebrospinal fluid (CSF) neopterin (NPT) levels with prognosis.

**Methods:**

The records of children with viral encephalitis who received blood purification treatment in the authors’ hospital from September 2019 to February 2022 were retrospectively analysed. According to the blood purification treatment mode, they were divided into the experimental group (HP + CVVHDF, 18 cases), control group A (CVVHDF only, 14 cases), and control group B (16 children with mild viral encephalitis who did not receive blood purification treatment). The correlation between the clinical features, severity of the disease and the extent of lesions on brain magnetic resonance imaging (MRI) and the CSF NPT levels was analysed.

**Results:**

The experimental group and control group A were comparable with respect to age, gender and hospital course (*P* > 0.05). There was no significant difference in speech and swallowing functions between the two groups after treatment (*P* > 0.05) and no significant difference in 7 and 14-day mortality (*P* > 0.05). The CSF NPT levels in the experimental group before treatment were significantly higher compared with control group B (*P* < 0.05). The extent of brain MRI lesions correlated positively with CSF NPT levels (*P* < 0.05). In the experimental group (14 cases), the serum NPT levels decreased after treatment, whereas the CSF NPT levels increased after treatment, and the differences were statistically significant (*P* < 0.05). Dysphagia and motor dysfunction correlated positively with CSF NPT levels (*P* < 0.05).

**Conclusion:**

Early HP combined with CVVHDF in the treatment of severe viral encephalitis in children may be a better approach than CVVHDF only for improving prognosis. Higher CSF NPT levels indicated the likelihood of a more severe brain injury and a greater possibility of residual neurological dysfunction.

## Introduction

Viral encephalitis in children is caused by the invasion of the brain by different viruses, resulting in acute diffuse inflammation of the brain parenchyma. It is a common infectious disease of the nervous system in children [1]. Severe viral encephalitis is characterised by acute onset, severe illness and rapid progression, which is often accompanied by multiple organ dysfunction syndrome. Viral encephalitis is a severely debilitating disease with high mortality. During viral encephalitis, the blood–brain barrier is disrupted and immune cells enter the central nervous system, triggering an immune response [2]. Non-regenerative neuronal tissue damage caused directly by infection or indirectly by the inflammatory response can lead to long-term neurological dysfunction [3, 4]. Recent studies that have developed more extensive neuropsychological testing and quality of life (QOL) assessments have shown that many patients continue to have ongoing cognitive, behavioral, or psychological deficits years after their first episode [5]. The resulting high disability rate seriously affects the child's health and quality of life and places a significant burden to their family and society [6]. Traditional treatment regimens have little effect on treating the disease and a slow onset. Hormones and human immunoglobulins have been used for the immune response, but their efficacy is uncertain.

Neopterin (NPT) is a low-molecular-weight compound derived from guanosine triphosphate metabolism in the human body and is primarily synthesised by monocytes/macrophages [7]. It is biologically stable and has been used for the diagnosis, differential diagnosis and prognosis evaluation of certain diseases [8]. Cerebrospinal fluid (CSF) NPT is a sensitive marker of inflammation that reflects secretion of microglia in the myelin sheath and can accurately detect inflammation in the central nervous system [9–11]. Accordingly, the detection of NPT in CSF is extremely important for the evaluation of infectious diseases of the central nervous system, particularly severe viral encephalitis.

A recent study reported for the first time that NPT in CSF was significantly elevated (> 30 nmol/L) in children with influenza-associated encephalopathy and may act as a valuable biomarker for its diagnosis. However, whether CSF NPT levels could be used as a prognostic marker requires further study [12].

Blood purification technology has been widely used in critically ill children and can effectively remove inflammatory mediators and cytokines [13, 14]. Continuous haemofiltration is effective for removing small and medium-sized harmful molecular substances from the blood, but its efficacy in removing endotoxins and macromolecular inflammatory cytokines is poor. In contrast, haemoperfusion (HP) is significantly better than haemofiltration for reducing concentrations of various medium-sized molecules, including inflammatory cytokines. Therefore, combining the advantages of the two different blood purification modes can effectively reduce the levels of cytokines in the blood of children and reduce the damage to organ function caused by inflammatory mediators [15].

Presently, few domestic studies have been conducted on the effect of HP combined with continuous venovenous haemodiafiltration (CVVHDF) in the treatment of severe viral encephalitis in children. Neopterin levels in the CSF of children with viral encephalitis have also not been studied. The present study was conducted to explore whether HP combined with the CVVHDF mode was advantageous in the treatment of severe viral encephalitis in children. The effect of this integrated blood purification mode on the levels of NPT in the CSF and serum of these children was analysed and the correlation between the CSF NPT levels and prognosis were reviewed.

## Patients and methods

### Patients

The clinical data of children with severe viral encephalitis who had been treated at the Children's Intensive Care Unit. the First Affiliated Hospital of Xinxiang Medical University from September 2019 to February 2022 were retrospectively analysed. A total of 48 children were included. Among them, 18 patients received HP + CVVHDF (the experimental group), 14 received CVVHDF only (control group A), and 16 patients with mild viral encephalitis who had not received blood purification treatment (control group B).

In the experimental group, 1 child had an influenza A virus infection, 1 had a varicella-zoster virus infection and 3 were infected with herpes simplex virus type 1 (HSV-1). No pathogen could be identified in the remaining patients. In control group A, 4 children had an enterovirus 71 (EV71) infection; the rest had no identifiable pathogen. In control group B, there were 3 children with an EV71 infection and 1 with HSV-1; the rest had no identifiable pathogen. The informed consent of the guardians of the children was obtained before their inclusion in the study. The study protocol was approved by the Ethics Committee of the First Affiliated Hospital of Xinxiang Medical University (approval number, 2,019,046).

Patients who met the diagnostic criteria for paediatric viral encephalitis were included in the study [16]. Those who met one of the following conditions were classified as severe cases: 1) acute encephalopathy following a prodromal infection, coma or severe brain dysfunction; 2) convulsive status; 3) respiratory failure; 4) circulatory failure; 5) associated failure of other organs; 6) cranial magnetic resonance imaging (MRI) findings indicating symmetrical and bilateral multifocal brain injury in the thalamus, periventricular white matter, internal capsule, putamen, dorsal upper brainstem and cerebellar medulla.

The following patients were excluded from the study: children with immunodeficiency, congenital heart disease, inherited metabolic disease, other infections of the central nervous system and those with incomplete clinical data.

## Methods

Routine treatment of all patients included mechanical ventilation where required, broad-spectrum antiviral therapy, measures for intracranial pressure reduction, anti-convulsants, sedatives and analgesics, nutritional support, glucocorticoids combined with human immunoglobulin and other supportive therapies as indicated.

Control group B received only conventional treatment; control group A received CVVHDF in addition to conventional treatment. The experimental group received HP combined with CVVHDF alongside conventional treatment. The size of the blood filter used was based on the weight of the child, and the femoral vein was cannulated in all children.

The applied treatment times were as follows: 1 day/time, each time CVVHDF ≥ 6 h/d, CVVHDF ≥ 3 times; 1 day/time, each time HP ≥ 2 h/d, CVVHDF ≥ 6 h/d, HP combined with CVVHDF ≥ 3 times. The following settings were used: blood flow velocity, 3 ÷ 5 ml/kg/min; replacement fluid rate, 30 ÷ 45 ml/kg/min; and ultrafiltration rate 3 ÷ 5 ml/kg/h (depending on circulation and fluid load). Citric acid (4%; 8 g in 200 ml) was used for in vitro anticoagulation.

The following data were extracted from the records: the patient’s age, gender, weight, pre-hospital course of disease, coma time, length of paediatric intensive care unit (PICU) stay, mechanical ventilation time, whether coma time was more or less than 7 days, the presence of convulsions (if any), cranial MRI findings, duration of mechanical ventilation, total hospital stay and mortality at 7 and 14 days. The Glasgow Coma Scale (GCS) was assessed by two experienced attending physicians in the acute (within 7 days) and the recovery (within 14 days) stages. Patients were followed up for two months to review any changes in neurological status (motor, speech, and swallowing functions) and were evaluated by professional rehabilitation physicians. Serum and cerebrospinal fluid samples were collected, and neopterin concentrations in serum and cerebrospinal fluid were measured and recorded at the Life Science Research Center of the First Affiliated Hospital of Xinxiang Univeristy. The NPT levels were not determined for four patients in the experimental group. In the remaining 14 patients, NPT levels in serum and CSF were evaluated before and after blood purification treatment; NPT levels were not evaluated for any of the patients in control group A. In control group B, NPT levels were evaluated only in the CSF and only before treatment.

### Statistical analysis

Statistical analysis was conducted using the SPSS Statistics for Windows 25.0. (IBM, Armonk, NY) and GraphPad Prism (v.8) software packages. Measurement data were expressed as median and interquartile range (IQR), and enumeration data were expressed as numbers and percentages (n, %). A chi-squared test or Fisher's exact test was used to compare two groups, and a *P* < 0.05 was considered statistically significant. The Spearman correlation coefficient was used to study the correlation between the extent of lesions in the cranial MRI and CSF NPT levels. The Spearman correlation coefficients were also used to study the correlation between disease severity and CSF NPT levels. A *P* value < 0.05 was considered statistically significant. A positive value for (‘r’) indicated a positive correlation whereas a negative value for ‘r’ indicated a negative correlation.

## Results

A total of 32 children diagnosed with severe viral encephalitis were included in this study (18 in the experimental group and 14 in control group A). There was no significant difference in age, gender, body weight, pre-hospital course of disease, PICU stay time, mechanical ventilation time, coma time (> 7 days or < 7 days), convulsions and total hospital stay between the two groups (*P* > 0.05) (Table 1).

**Table 1:** Clinical data of two groups of children

Clinical information	Control group A (*n* = 14)	Experimental group (*n* = 18)	Z value	*P* value
Age (year)	6.50 (1.75,11.00)	9.00 (6.00,12.50)	-1.775	0.076
Sex				
Male,n (%)	8 (57.14)	10(55.56)		1.00
Female,n (%)	6 (42.86)	8 (44.44)		
Weight (kg)	20.5. (10.00,38.00)	40.00 (20.75,56.50)	-1.845	0.065
Pre-hospital course (days)	3.00 (1.75,4.25)	3.00 (1.75,4.25)	-0.019	0.985
PICU stay time (days)	12.00 (7.75,18.75)	16.50 (8.50,22.00)	-0.799	0.424
Mechanical ventilation time (days)	7.00 (4.50,12.00)	6.00 (4.00,10.00)	-0.706	0.480
Coma time				
< 7 days	9 (64.29%)	12 (66.67%)		1.00
> 7 days	5 (35.71%)	6 (33.33%)		
Twitch				
Yes (%)	10 (43.50)	13 (56.50)		1.00
No (%)	4 (44.4)	5 (55.60)		
Total hospital stay (days)	34.50 (16.50,76.75)	31.50 (16.50,64.00)	-0.152	0.879

### Comparison of neurological function and prognosis between the experimental group and control group A after treatment

Following treatment, the GCS scores in both the experimental and control group A showed considerable improvement compared with before treatment, and the differences were statistically significant (*P* = 0.000412 and *P* = 0.0003131, respectively) (Table 2). There was no significant difference in GCS scores before and after treatment between the two groups (*P* > 0.05). The motor function of the children in both the experimental and control group A significantly improved after treatment, and the difference was statistically significant (*P* = 0.031) (Table 3). There was no mortality at 7 and 14 days in either the experimental or control groups A (see Table 4).

**Table 2:** Comparison of Glasgow scores between the two groups before and after treatment

Group	Glasgow score before treatment	Glasgow score after treatment	Z value	*P* value
Control group (*n* = 14)	5.50 (3.00,8.00)	10.50 (6.00,13.00)	-2.955	0.0003131^***^
Experimental group (*n* = 18)	6.00 (3.00,6.50)	10.00 (6.75,15.00)	-3.532	0.000412^***^
Z value	-0.11756	-0.650		
*P value*	0.906	0.516		

^***^ means *P* < 0.001

**Table 3:** Comparison of motor, speech and swallowing functions in the two groups after treatment

	Movement disorder		*P* value	Speech disorder		*P* value	Dysphagia		*P* value
	Yes	No		Yes	No		Yes	No	
Control group (*n* = 14)	10	4	0.031*	7	7	0.473	5	9	0.712
Experimental group (*n* = 18)	5	13		6	12		5	13	

^*^ represents *P* < 0.05

**Table 4:** Comparison of the prognosis of the two groups of children

Relevant factors		7-day death cases (%)	14-day death cases (%)
Control group	(*n* = 14)	0 (0)	0 (0)
Experimental group	(*n* = 18)	0 (0)	0 (0)

### Comparison of cerebrospinal fluid neopterin levels between the experimental group (14 cases) and control group B (16 cases) and the correlation between the extent of lesions on brain magnetic resonance imaging and the cerebrospinal fluid neopterin levels in the two groups

**Fig. 1:**
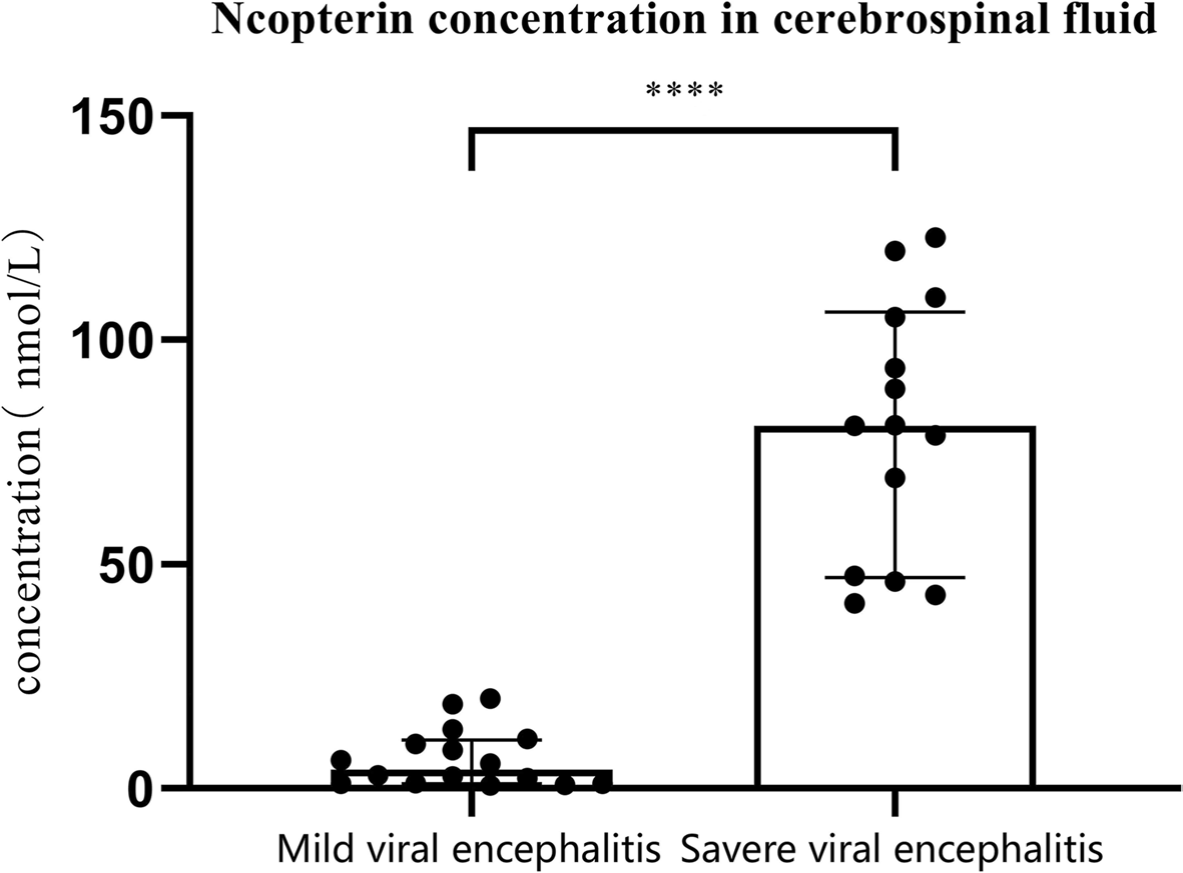
Comparison of Neopterin levels in cerebrospinal fluid of two groups of children. Note: **** means *P* < 0.0001

Before treatment, the CSF NPT levels in the experimental group were significantly higher than in control group B, and the difference was statistically significant (*P* < 0.0001) (Fig. 1). The Spearman correlation analysis between control group B and the experimental group (14 cases) showed a positive correlation between the extent of lesions on brain MRI and CSF NPT levels (*P* = 0.00029) (Table 5).

**Table 5:** Correlation analysis between the involved sites of head MRI and the level of Neopterin in cerebrospinal fluid in mild and severe viral encephalitis

Relevant factors	Neopterin levels in cerebrospinal fluid	
	r	*P*
Cranial MRI Involvement	0.686	0.00029^***^

^**^ means *P* < 0.01

### Comparison of neopterin levels in serum and cerebrospinal fluid before and after treatment in the experimental group

Following the integrated blood purification treatment, the NPT levels in serum were significantly lower than before treatment. However, the CSF NPT levels after treatment were higher than before treatment, and the difference was statistically significant (*P* < 0.05) (Table 6).

**Table 6:** Comparison of Neopterin in serum and cerebrospinal fluid before and after treatment

Laboratory Metrics	Before treatment (*n* = 14) nmol/L	After treatment (*n* = 14) nmol/L	T value	*P* value
Np in serum	29.61 ± 9.02	20.81 ± 8.63	5.668	< 0.0001^****^
Np in cerebrospinal fluid	80.56 ± 28.21	92.05 ± 33.42	-2.302	0.038*

### The correlation between clinical features, disease severity, nervous system function and cerebrospinal fluid neopterin levels in critically ill children

Following integrated blood purification treatment, swallowing dysfunction showed a positive correlation with CSF NPT levels, and this was statistically significant (*P* < 0.05). Similarly, motor dysfunction showed a positive correlation with CSF NPT levels, and this was statistically significant (*P* < 0.05) (Table 7).

**Table 7:** Correlation of clinical data, disease severity, nervous system function and neopterin level in cerebrospinal fluid in children

	Before therapy		After therapy	
	r	*P*	r	*P*
Age	0.116	0.692	0.271	0.349
Sex	0.478	0.084	0.301	0.295
Weight	0.136	0.642	0.414	0.142
Glasgow score	-0.471	0.089	-0.277	0.338
Cranial MRI Involvement	-0.405	0.151	0.051	0.864
Mechanical ventilation time	0.161	0.582	0.219	0.452
Coma time	0.102	0.727	0.062	0.832
Sports function	0.367	0.197	0.713	0.004^**^
Speech function	0.388	0.170	0.499	0.069
Swallowing function	0.628	0.016^*^	0.706	0.005^**^

^*^ represents *P* < 0.05, ** represents *P* < 0.01

## Discussion

In this study, the outcomes of patients receiving integrated blood purification (HP + CVVHDF) treatment with conventional treatment were compared with those who received only the CVVHDF and conventional treatment. It was found that the efficacy of HP combined with CVVHDF in the treatment of children with severe viral encephalitis was better than using CVVHDF only, particularly for improving motor function.

There was no significant difference in the GCS scores, speech function, swallowing function, 7-day and 14-day mortality between the experimental group and control group A. The results of this study showed that the GCS score improved significantly after treatment in two groups, i.e. those who had received HP + CVVHDF, as well as those who had received CVVHDF only. This was consistent with existing studies. Liu Min et al. [17] found that the GCS scores of children with severe viral encephalitis treated by continuous blood purification were better than those who received traditional treatment only. Ni Jingwen et al. [18] also found that blood purification in children with influenza-related nervous system injuries could result in improvement of the GCS score and nervous system function and relieve intracranial hypertension.

Compared with children with mild viral encephalitis (control group B), the CSF NPT levels in children with severe viral encephalitis (the experimental group and control group A) were significantly higher. A correlation between NPT concentration in CSF and the CSF viral load was previously described [19]. Molero-Luis M et al. [20] found that CSF NPT levels higher than 61 nmol/L were helpful for distinguishing inflammatory and non-inflammatory neurological diseases but could not identify the aetiology of neurological disease. They also found that CSF NPT levels were significantly increased in inflammatory neurological diseases caused by both bacterial and viral infections.

This study found that the extent of lesions on brain MRI in mild and severe viral encephalitis correlated positively with CSF NPT levels. Magnetic resonance imaging can detect the location of a specific lesion and its size. It is highly intuitive and has important reference value for the diagnosis and prognosis evaluation of children [21]. Studies have shown that CSF NPT levels are associated with the presence of inflammatory lesions on MRI scans [22]. The more abnormal an MRI scan appears for children with viral encephalitis, the more severe the disease will likely be. In this study, the CSF NPT levels were significantly higher in children for whom more extensive lesions were observed on brain MRI scans.

It has been reported that viral infections cause an immune response resulting in the production of inflammatory factors, which, in turn, accelerate the progression of the disease and cause major organ dysfunction [23]. Continuous blood purification is beneficial for reducing the levels of inflammatory mediators, thereby preventing the inflammatory response cascade [24]. Little is known about the efficacy of multiple blood purification modalities in children with severe viral encephalitis and the association of NPT levels with prognosis. Putz et al. [25] found that patients with end-stage renal disease had decreased plasma levels of NPT each time they underwent haemodialysis, and that the concentration of NPT in patients with end-stage renal disease was related to the length of haemodialysis treatment time [26]. Sadeghi et al. [27] also found that the plasma NPT concentrations in renal transplant patients decreased significantly after plasma exchange treatment.

This study found that NPT levels in serum significantly decreased after integrated blood purification treatment for severe viral encephalitis, while the levels in CSF were not affected by changes in the serum concentration. This result was corroborated in another study [28].

The main purpose of the present study was to observe the efficacy of HP combined with CVVHDF in the treatment of severe viral encephalitis in children. While CVVHDF only was effective for removing small and medium-sized harmful molecular substances in the blood, its effectiveness for removing endotoxins and inflammatory cytokines of larger molecular size was poor. In contrast, HP was significantly more successful than hemofiltration for removing concentrations of various medium-sized molecules, including inflammatory cytokines. Using the advantages of two different blood purification modes to complement one another, it is possible to reduce the level of cytokines in the blood and thereby reduce the damage to organ function caused by inflammatory mediators [15]. The results of the current study showed that the improved outcome may have been related to the removal of inflammatory mediators in the body by blood purification technology. A possible mechanism for the better outcome after blood purification may be a decrease in demyelination of the nervous system and cerebrovascular and perivascular damage induced by the inflammatory response. At present, there are few studies on the combination of two or more blood purification modes in the treatment of children with critical illnesses. Further research is thus needed in this regard.

Both swallowing and motor dysfunction correlated positively with NPT levels in CSF before and after treatment. According to Ghisoni et al. [29], neopterin is not an inert compound but a cytoprotective molecule synthesised and secreted by nerve cells as a response to injury or inflammation. That is, the more severe the brain damage or inflammatory response, the more neopterin will be synthesised and secreted by nerve cells. This study showed that the more severe the sequelae in the recovery period, the higher the CSF NPT levels will be. The correlation between disease prognosis and NPT levels requires further confirmation by a prospective study using a larger sample size.

This study includes some shortcomings, i.e. it did not continuously monitor the CSF NPT levels of the children during the recovery period. Furthermore, the CSF NPT levels were not monitored long-term to study their correlation with nervous system functions (motor function, language function, and swallowing function). Moreover, this research was a single-centre study, the conclusions of which require further confirmation by a large number of prospective studies.

## Conclusion

Early HP combined with CVVHDF in the treatment of severe viral encephalitis in children may be a better approach than single CVVHDF for improving prognosis. The higher the level of NPT in the CSF of children with viral encephalitis, the more severe the brain injury may be, and the greater the possibility of residual nervous system dysfunction.

## Data Availability

All data generated or analyzed during this study are included in this published article.

## Abbreviations

HP: Hemoperfusion
CVVHDF: Continuous veno venous hemodiafiltration
CSF: Cerebrospinal fluid
NPT: Neopterin
MRI: Magnetic resonance imaging
QOL: Quality of life
EV71: Enterovirus
HSV-1: Herpes simplex virus type 1
PICU: Paediatric intensive care unit
GCS: Glasgow Coma Scale
ELISA: Enzyme-linked immunosorbent assay
IQR: Interquartile range
